# The Role of Acrolein and NADPH Oxidase in the Granulocyte-Mediated Growth-Inhibition of Tumor Cells

**DOI:** 10.3390/cells8040292

**Published:** 2019-03-29

**Authors:** Morana Jaganjac, Tanja Matijevic Glavan, Neven Zarkovic

**Affiliations:** 1 Department of Molecular Medicine, Rudjer Boskovic Institute, HR-10002 Zagreb, Croatia; mjaganjac@adlqatar.qa (M.J.); tmatijev@irb.hr (T.M.G.); 2 Anti-Doping Lab Qatar, Life Science and Research Division, Doha 27775, Qatar

**Keywords:** reactive oxygen species (ROS), oxidative stress, lipid peroxidation, acrolein, 4-hydroxynonenal (4-HNE), oxidative burst, granulocytes, cancer cells, growth control, cancer regression

## Abstract

Although granulocytes are the most abundant leukocytes in human blood, their involvement in the immune response against cancer is not well understood. While granulocytes are known for their “oxidative burst” when challenged with tumor cells, it is less known that oxygen-dependent killing of tumor cells by granulocytes includes peroxidation of lipids in tumor cell membranes, yielding formation of reactive aldehydes like 4-hydroxynonenal (4-HNE) and acrolein. In the present work, we investigate the role of reactive aldehydes on cellular redox homeostasis and surface toll-like receptor 4 (TLR4) expression. We have further study the granulocyte-tumor cell intercellular redox signaling pathways. The data obtained show that granulocytes in the presence of 4-HNE and acrolein induce excessive ROS formation in tumor cells. Acrolein was also shown to induce granulocyte TLR4 expression. Furthermore, granulocyte-mediated antitumor effects were shown to be mediated via HOCl intracellular pathway by the action of NADPH oxidase. However, further studies are needed to understand interaction between TLR4 and granulocyte-tumor cell intercellular signaling pathways.

## 1. Introduction

Granulocytes are the most abundant leukocytes in the human body, which provide the first line of defense against pathogens and play a fundamental role in the innate immune response [1]. Although granulocytes are closely associated with tumor cells, their exact role within tumor microenvironment remains controversial [2,3,4]. Activated granulocytes generate vast amounts of reactive oxygen species (ROS) that were shown to be crucial for the oxygen-dependent killing of tumor cells [5,6]. ROS generation by activated granulocytes involves various mechanisms, among which NADPH oxidase seems to be the key source of granulocyte derived ROS during the oxidative burst [7,8]. Activation of NADPH oxidase was shown to be toll-like receptor 4 (TLR4)-dependent in response to hemorrhagic shock or pathogens [9,10]. Over a decade ago it was suggested that a tumor cell-induced oxidative burst of granulocytes could also be via activation of toll-like receptors (TLR) [5], however this still remains to be elucidated.

Depending on the amount of ROS and their distance from the target cells, ROS can mediate cellular responses or cause oxidative damage to macromolecules [3,11,12]. Bauer has earlier described four ROS intercellular signaling pathways, among which the induction of apoptosis of transformed cells was mainly via HOCl intercellular signaling pathways [13]. In granulocyte-mediated tumor cell destruction, this pathway would depend on the superoxide anion (^•^O_2_^−^) derived from both sides. The granulocyte NADPH oxidase generates ^•^O_2_^−^ that further dismutase to hydrogen peroxide (H_2_O_2_). The H_2_O_2_ is then converted into HOCl by the action of myeloperoxidase (MPO). HOCl can also react with ^•^O_2_^−^, yielding highly reactive hydroxyl radical (OH^•^), that is able to directly induce lipid peroxidation (LPO) [3].

Peroxidation of polyunsaturated fatty acids (PUFAs) that are esterified in membranes can yield reactive aldehydes, such are acrolein and 4-hydroxynonenal (4-HNE). High amounts of reactive aldehydes are cytotoxic. However, in small amounts they have important cell signaling roles of regulating cell proliferation, differentiation, and apoptosis. Reactive aldehydes are longer living molecules, compared to ROS, that alter different signaling pathways either directly or indirectly by covalent modification of macromolecules [14,15,16]. Reactive aldehydes can also be generated by activated granulocytes that employ an MPO hydrogen peroxide chloride system that can convert L-threonine into 2-hydroxypropanal, an acrolein precursor, and acrolein [17]. Although the involvement of reactive aldehydes in tumor development has been intensively studied for decades, the exact mechanisms of their concentration dependents and cell type specific effects on the tumor–host relationship need to be further elucidated [18,19,20,21,22,23].

Therefore, in the present work we investigate the involvement of two particular reactive aldehydes, acrolein, being the most reactive, and 4-HNE, a major biomarker of lipid peroxidation also denoted as “second messenger of free radicals”, on the respiratory burst of granulocytes, as well as the granulocyte-cancer cell intercellular signaling, in order to reveal the critical steps responsible for granulocyte-mediated destruction of cancer cells.

## 2. Materials and Methods

### 2.1. Animals

Experiments were performed on three-month-old male Sprague Dawley rats. Water and food were given ad libitum. All the experiments were performed in accordance with the ILAR Guide for the Care and Use of Laboratory Animals, EU Council Directive (86/609/EEC) and the Croatian Animal Protection Act (Official Gazette 135/06). Ethical approval obtained from the RBI and MSES for the grant MSES 098-0982464-2519.

### 2.2. Treatment of Animals and Isolation of Granulocytes

As a source for granulocytes, 4 healthy control rats were used. Rats were s.c. injected with 5 mL Sephadex on each side, in the lower dorsal quadrant, and 24 h later the Sephadex papules were surgically removed and the granulocytes were isolated, as described before [5]. The granulocyte cell suspension was counted and diluted in a RPMI 1640 medium to the desired concentration, while their viability, checked by the trypan blue exclusion test, was found to be 97%.

### 2.3. Tumor Cell Lines

Three rat derived cell lines, C6 glioma, Walker W256 carcinoma, and PC12 rat adrenal pheochromocytoma, (ATCC, CCL-107, CCL-38, CRL-1721) were used. The cells were adapted to grow as monolayers cultured at 37 °C in a RPMI 1640 medium, supplemented with 5% (W256 and PC12) or 10% (C6) fetal bovine serum (FBS) and 1% Penicillin/Streptomycin in a humidified atmosphere.

### 2.4. 4-HNE and Acrolein

The aldehyde, in the form of 4-hydroxy-2-nonenal-dimethylacetal (HNE-DMA), was kindly provided by the Institute of Molecular Biology, Biochemistry and Microbiology, Graz, Austria. Prior to the experiment, it was activated with 1 mM HCl (Kemika, Zagreb, Croatia) for 2 h. The concentration of HNE was determined by spectrophotometry (UV-1601 spectrophotometer; Shimadzu, Tokyo, Japan) [24].

Prior to treatment, cells were left in a serum free medium for 1 h. HNE and acrolein were freshly prepared before each experiment and used in a concentration of 12.5 µM in a RPMI medium (2% FCS).

### 2.5. Investigating the Ability of 4-HNE and Acrolein to Modulate Granulocyte and Tumor Cells Redox Homeostasis

Intracellular ROS production was examined in granulocytes and tumor cells using a redox sensitive probe 2,7-dichlorodihydrofluorescein diacetate (DCFH-DA; Fluka, Steinheim, Germany), as described before [25,26]. Briefly, the tested cells (granulocytes or tumor cells) were treated with 10 µM DCFH-DA in Hanks’ Balanced Salt Solution (HBSS) in 5% CO_2_/95% air at 37 °C for 30 min. Afterwards the DCFH loaded cells were either co-cultured with the other type of cells (tumor cells or granulocytes, respectively) or left alone as control. The seeding density of the granulocytes and tumor cells, cultured in 96-well microcytoplates, was 2 × 10^5^ and 2 × 10^4^ cells per culture, respectively. The mixed cultures were then washed and incubated with an HBSS buffer, supplemented with 12.5 μM HNE, 12.5 μM acrolein, or left untreated as control. After 2 h, the fluorescence intensity was measured with a Variant fluorescence spectrophotometer with an excitation wavelength of 500 nm and emission detection at 529 nm. The arbitrary units, relative fluorescence units (RFU), were based directly on fluorescence intensity.

### 2.6. Determination of Surface TLR4 Expression by Flow Cytometry

Influence of reactive aldehydes on the TLR4 expression was measured in granulocytes and W256 tumor cells. The seeding density of granulocytes, cultured in 24-well plates, was 10^6^ cells per culture in the RPMI 1640 medium. The seeding density of the tumor cells was 2 × 10^5^ cells per culture, irrespective if cultured alone or if added to the granulocytes. The cell cultures were incubated for 2 h in the RPMI 1640 medium, supplemented with 12.5 μM HNE, 12.5 μM acrolein, or left untreated as control, at 37 °C in a humidified air atmosphere with 5% CO_2_. Cells were then washed with PBS and incubated with an anti-TLR4 antibody (Abcam, Cambridge, UK) or an isotype control (Abcam, UK) for 60 min, followed by the incubation with an AlexaFluor 488 conjugated secondary antibody (Invitrogen, Carlsbad, CA, USA) for 60 min on ice. The stained cells were then analyzed with FACSCalibur™ (Becton Dickinson, San Jose, CA, USA). The results were analyzed with WinMDI v2.9 software (Joseph Trotter, San Diego, CA, USA).

### 2.7. The Effect of Granulocyte Intercellular HOCl Signaling on the Tumor Cell Proliferation

Granulocytes were co-cultured with tumor cells at a ratio of 10:1 in 96-well plates and the proliferation of C6 and PC12 tumor cells was measured by the ^3^H-thymidine (^3^H-TdR) incorporation assay, similarly to as described before [27]. The effects of HOCl intercellular redox signaling on tumor cell proliferation were investigated using the NADPH oxidase inhibitor apocynin (APO, 50 µg/mL, Sigma, Steinheim, Germany), the peroxidase inhibitor 4-aminobenzoyl hydrazide (ABH, 25 µM, Acros Organics, Geel, Belgium), the HOCl scavenger taurine (Tau, 25 mM, Sigma), the hydroxyl radical scavenger mannitol (Man, 10 mM, Kemika, Zagreb, Croatia), or the singlet oxygen scavenger histidine (2 mM, Sigma). Briefly, tumor cells alone or co-cultured with granulocytes were exposed to different inhibitors and incubated for 24 h at 37 °C in a humidified air atmosphere with 5% CO_2_. After 24 h, radioactive ^3^H-TdR was added to each culture and left for additional 48 h. The incorporation of ^3^H-TdR was measured by a β-liquid-scintillation counter (LS 3800 Series; Beckman, Indianapolis, IN, USA).

### 2.8. Statistics

Descriptive statistics were shown as the mean ± SD. The significance of differences between groups was assessed using the Student *t*-test and the Chi-square test. When more than two groups were compared, we used one sided ANOVA with appropriate post hoc testing. The IBM SPSS Statistics v21 (IBM Corp., New York, NY, USA) software for Microsoft Windows were used. Differences with *p* less than 0.05 were considered as statistically significant.

## 3. Results

**Figure 1:**
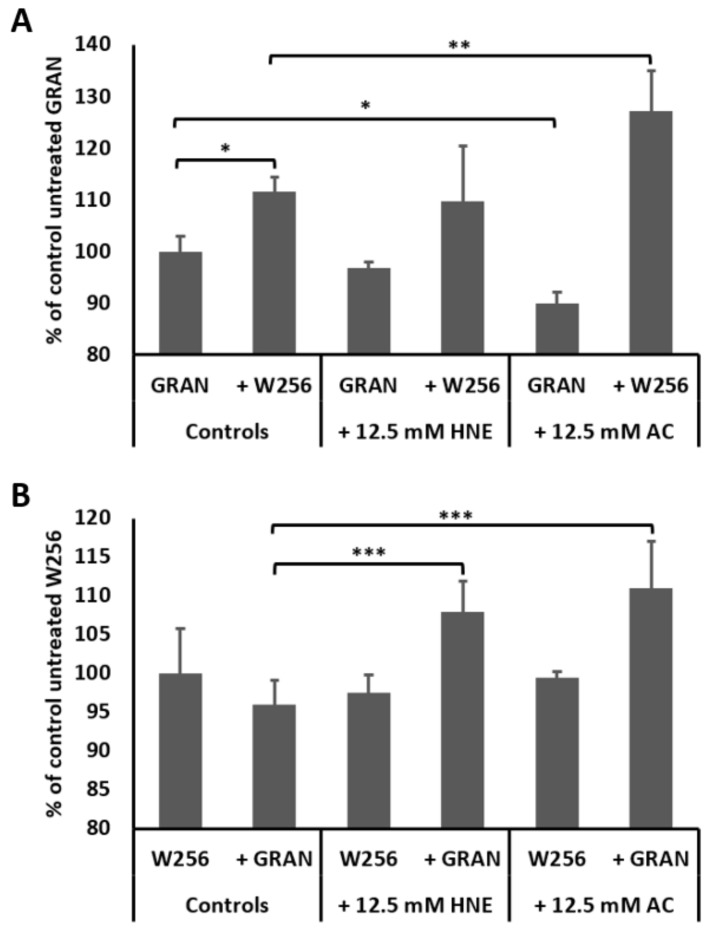
Intracellular ROS production in granulocytes (**A**) and in W256 tumor cells (**B**). Mean values ± SD are given, (*) significance *p* < 0.05 compared to untreated granulocytes, (**) significance *p* < 0.01 compared to co-culture of granulocytes and W256 tumor cells and (***) significance *p* < 0.05 compared to co-culture of granulocytes and W256 tumor cells.

Intracellular ROS production in granulocytes and W256 tumor cells is presented in Figure 1. Exposure of granulocytes to reactive aldehydes 4-HNE and acrolein did not stimulate granulocyte intracellular ROS production, while acrolein itself even reduced granulocyte intracellular ROS production when compared to untreated granulocytes (*p* < 0.05). However, in the presence of W256 tumor cells, granulocytes showed a significant increase of the intracellular ROS production. Such increment of the oxidative burst of granulocytes was further enhanced in the presence of acrolein (Figure 1A, *p* < 0.05), but not in the presence of 4-HNE (Figure 1A, *p* > 0.05). Granulocytes themselves did not influence intracellular ROS production in W256 cancer cells (Figure 1B, *p* > 0.05), while the addition of both reactive aldehydes caused a significant increase of intracellular ROS production by cancer cells (Figure 1B, *p* < 0.05, for both 4-HNE and acrolein).

**Figure 2:**
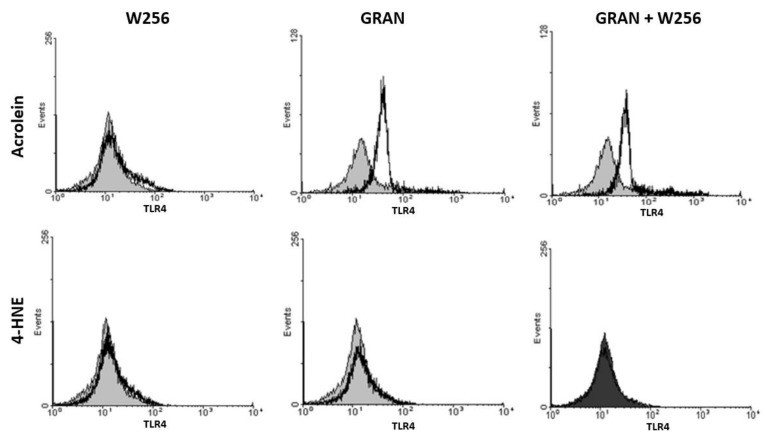
Representative flow cytometry histograms showing TLR4 surface expression on granulocytes and W256 tumor cells. Granulocytes and tumor cells were stained with antibodies specific for TLR4 (open histogram) or their isotype control (gray histogram).

The impact of 4-HNE and acrolein on the TLR4 surface expression of granulocytes and of W256 cancer cells is shown in Figure 2. Although 4-HNE did not show any particular effect on the TLR4 expression, a significant shift was observed when granulocytes were exposed to acrolein, regardless of the presence of tumor cells (Figure 2).

**Figure 3:**
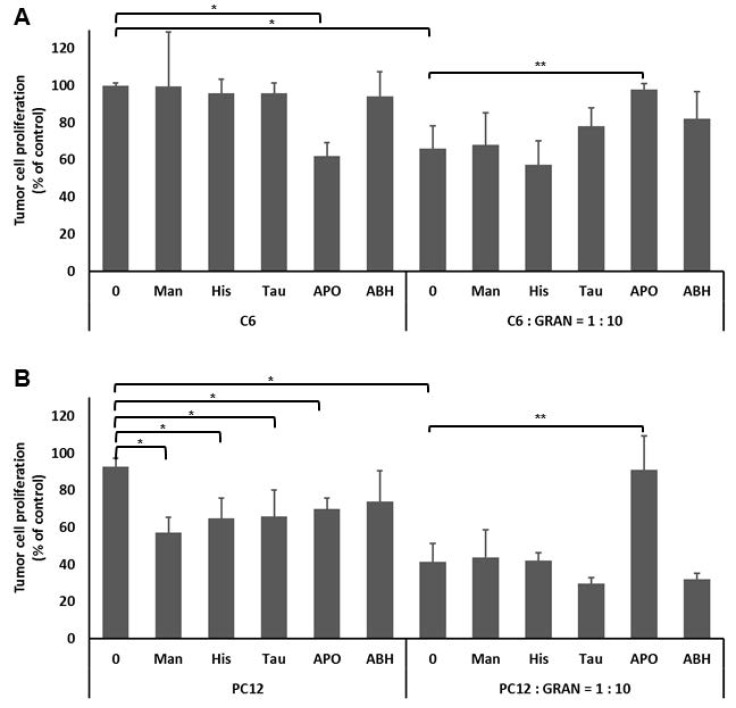
Granulocyte HOCl intercellular redox signaling inhibits tumor cell proliferation. C6 (**A**) or PC12 (**B**) tumor cells treated with granulocytes in the presence or absence of inhibitors (Man—hydroxyl radical scavenger; Tau—HOCl scavenger; His—singlet oxygen scavenger; APO—NADPH oxidase inhibitor; ABH—peroxidase inhibitor). Mean values ± SD are given, (*) significance *p* < 0.01, compared to untreated tumor cells, (**) significance *p* < 0.01 compared to co-culture of granulocytes and tumor cells, and *p* > 0.01 compared to tumor cells alone.

Since the anticancer effects of granulocytes were already well studied on W256 cancer cells, the effect of granulocytes on the proliferation of the other murine cancer cells, notably on PC12 pheochromocytoma and C6 glioma, were studied for the first time, as is shown in Figure 3. Granulocytes inhibited the proliferation by 40% and 55% for C6 and for PC12 tumor cells, respectively. In order to understand the impact of specific granulocyte derived ROS and the importance of intercellular redox signaling, the specific parts of intercellular HOCl signaling pathway were inhibited by the addition of respective inhibitors. Treatment of tumor cells with mannitol, histidine, taurine, and ABH did not have effect on C6 cell proliferation, while it reduced proliferation of PC12 cells. However, treatment of both C6 and PC12 cells with APO inhibited tumor cell proliferation compared to untreated tumor cells with the value *p* < 0.05. Furthermore, while the addition of mannitol, histidine, taurine, and ABH to the co-culture of granulocytes and tumor cells did not show any effect on the tumor cell proliferation, compared to untreated co-cultures (*p* > 0.05 for both cell lines), the addition of APO, which specifically inhibits the NADPH oxidase, abolished the anti-tumor effect of granulocytes (*p* < 0.05 for both C6 and PC12).

## 4. Discussion

The role of granulocytes in the host’s defense against cancer is not well understood, although the important role of granulocyte-derived ROS in the oxygen-dependent killing of tumor cells was recognized more than 30 years ago [28]. In the meantime, dual roles of granulocytes, both in tumor progression [5,6] and tumor regression [4,26,29], were documented. Since it is known that ROS generated by an oxidative burst of granulocytes can lead to production of reactive aldehydes acting as “second messengers of free radicals”, we hypothesize that reactive aldehydes in the presence of granulocytes might be the crucial factors determining the fate of cancer cells. In favor to our hypothesis are not only results of the current study, but also earlier studies, especially on human colon carcinoma [30]. Namely, production of 4-HNE by inflammatory and stromal cells increased autocrine/paracrine suppression of colon epithelial cells through TGF-β1 and c-Jun *N*-terminal kinase upregulation [31]. Complementary to that, acrolein was found to be associated with colon tumorigenesis, too. Namely, in cases of adenomas, the amount of acrolein was increased from a moderate appearance in tubular and villotubular low-grade adenomas to abundant and diffusive distribution in high-grade villotubular adenomas and Dukes A carcinomas. However, in advanced Dukes B and C carcinomas, acrolein was hardly noticed, while, opposite to cancer tissue, acrolein was abundant in non-malignant colon tissue adjacent to the cancer [32]. Though, it was initially assumed that such changes in reactive aldehydes, in particular of acrolein which is not usually present in human tissue under physiological circumstances, might reflect negative consequences of the food-derived LPO. Recent findings on human liver and primary, as well as metastatic, lung cancer suggest that reactive aldehydes might actually represent an important mechanism of host defense against cancer invasion [33,34]. Moreover, direct involvement of 4-HNE and acrolein was reported for granulocyte-mediated W256 regression. So, we may assume that, in addition to cases of human cancer, granulocytes might be the source of reactive aldehydes in the vicinity of cancer [28]. As the acrolein formation was observed in an early stage of W256 regression, we have proposed that acrolein might serve as a mediator of positive feedback to promote myeloperoxidase activity and further induce ROS production, eventually generating 4-HNE [29]. In the present work we show for the first time that reactive aldehydes, in the tested concentrations, did not solely affect in vitro cellular redox homeostasis, either of tumor cells or granulocytes. However, because acrolein enhanced the oxidative burst of granulocytes in the presence of W256 cells and vice versa, our current findings are in line with our earlier hypothesis. Although 4-HNE had similar effects in our study, the fact that it was less potent than acrolein might reflect differences in their biochemical reactivity and/or its recently reported inhibitory effect on the granulocyte activation and phagocytosis [35].

The most important role of reactive aldehydes might be in the disturbance of tumor cell redox homeostasis, as indicated in the current study, showing that only granulocytes in the presence of reactive aldehydes have the ability to significantly affect the redox balance of tumor cells. Namely, acrolein was shown to upregulate granulocyte surface TLR4 expression. However, whether the increase in TLR4 abundance on the membranes is the result of a shuttling of the protein or is an induction of the overall protein, still remains to be elucidated. Increased membrane expression of granulocyte TLR4 could impact specific signaling pathways, like NF-kappa B and STAT3, and could mediate molecular mechanisms to promote NADPH oxidase activation and, consequently, ROS production and growth suppression/decay of tumor cells [36]. Indeed, the granulocyte mediated antitumor effect was inhibited by APO, an NADPH oxidase inhibitor, but not by Man (a hydroxyl radical scavenger), Tau (a HOCl scavenger), His (a singlet oxygen scavenger), or ABH (peroxidase inhibitor). We have also observed that, despite the fact that APO blocked the anti-cancer effect of granulocytes, without the presence of granulocyte, APO was able to inhibit tumor cell proliferation. The inhibitory effect of APO has already been described earlier on both human and rat prostate cancer cell lines by inducing the cell cycle arrest [37,38]. In LNCaP prostate cancer cells, APO had an anti-proliferative effect only when added at higher concentrations, while it did not have effects at lower concentrations [38]. The above could explain the effects observed in this study, where addition of granulocytes increased the APO targets and decreased the chance of the inhibitor to exhibit its effects on tumor cells. Hence, our results indicate that granulocyte mediated malignant destruction is dependent on HOCl intercellular signaling pathways by the action of the NADPH oxidase. Similarly, recent study also highlighted the importance of the HOCl signaling pathway as the pathway that might prevent tumorigenesis in the lactobacillus/peroxidase system mediated antitumor effect [39].

The results of our study suggest two possible mechanisms of involvement of reactive aldehydes in the anti-cancer effects of granulocytes, as follows:

1. Reactive aldehydes, by impairing the membrane integrity, enable ROS to enter the tumor cells or
2. coordinated action of granulocyte derived ROS and reactive aldehydes can induce excessive intracellular ROS production, thus causing decay of cancer cells.

In conclusion, reactive aldehydes, formed as a consequence of an intense oxidative burst of granulocytes, might mediate antitumor effects of granulocytes by impairing tumor cell redox homeostasis and, also, at least in the case of acrolein, induce granulocyte TLR4 expression. The link between TLR4 expression and NADPH oxidase stimulated ROS generation is well known and might explain the granulocyte-mediated antitumor effects through the HOCl intracellular pathway by the action of the NADPH oxidase, which could eventually determine the tumor–host relationship in a desirable way for cancer regression.
